# Perceived economic situation, but not education level, is associated with disability prevalence in the Spanish elderly: observational study

**DOI:** 10.1186/1471-2318-14-60

**Published:** 2014-05-07

**Authors:** Angel Rodriguez-Laso, Antonio Abellan, Mayte Sancho, Rogelio Pujol, Ignacio Montorio, Purificación Diaz-Veiga

**Affiliations:** 1 Matia Instituto Gerontologico, San Sebastian, Spain; 2 Centre for Human and Social Sciences, Spanish National Research Council, Madrid, Spain; 3 Faculty of Psychology, Autonomous University of Madrid, Madrid, Spain

**Keywords:** Aged, Disabled persons, Social class, Prevalence, Spain

## Abstract

**Background:**

The aim of this paper is to ascertain if the subjective perception of the economic situation of a household is associated with the prevalence of disability in old age, net of education level. Subjective economic perception is less non-response biased. Knowing if the self-perceived economic situation is related to disability over and above education level has important implications both for understanding the mechanisms that lead to disability and for selecting policies to reduce it.

**Methods:**

This is a transversal study based on the pilot of the ELES survey, which is a representative survey of non-institutionalised Spaniards aged 50 and over. Only individuals whose job income levels were fixed before becoming disabled were selected to avoid the main source of reverse causality. Disability was defined as having difficulty in carrying out any of 12 activities of daily living. Education level, difficulty in making ends meet, self-perceived relative economic position of the household, age, gender, psychological disposition, and alcohol and tobacco consumption were introduced as independent variables in binary logistic models.

**Results:**

The working sample is made up of 704 individuals of aged 60 and over. The subjective household economic situation, measured in two different ways, is strongly and consistently related with the prevalence of disability net of age, gender, education level and psychological disposition. After adjusting for age and gender, education level is no longer associated with disability. However, having economic difficulties has the same effect on disability prevalence as being 10 years older, or being a woman instead of a man.

**Conclusions:**

As the economic situation of the elderly is much easier to improve than their formal education, our findings support feasible interventions which could lead to a reduction in the prevalence of disability.

## Background

Disability is a growing concern in societies all over the world which are getting older. Socioeconomic status has been found to be one of the most important determinants of disability among the general population [1-3] and the elderly [4-11]. Socioeconomic status is usually measured through education, occupation and income [12]. Education conditions occupation and therefore income during working life and afterwards. It could be argued that the effect of income on disability is due in fact to its association with education, which renders advantages in terms of knowledge, perceived control and social support [1,2]. However, it could also be argued that disposable income during adulthood and old age may have an effect independent of that of education level because it facilitates the purchase of preventive resources and care for already established diseases [3]. Proving one hypothesis or the other has important implications both for understanding the mechanisms that lead to disability and for supporting, or not, policies that transfer money from the richest to the poorest as a means of reducing the disability burden.

Of the three factors mentioned above, education, occupation and income, income is the most difficult to measure accurately. It is well-known that interviewees are not comfortable answering questions on the subject [12], especially when they have high incomes [13,14]. Missing data increase the chance of selection bias and hinder the interpretation of results. In addition, present income may be an inaccurate measure of the total financial resources available to an elderly person [14]. Alternatively, subjective economic position questions are less intrusive for respondents, who are less reluctant to answer them across the income spectrum, and can be a better measure of the total wealth of the household. Different alternatives have been employed in surveys: the possibility of having savings, financial hardship, difficulties in covering certain expenses, difficulties in making ends meet or financial satisfaction [15].

Another major problem when studying the effect of economic variables on disability is reverse causality. The main source of reverse causality in this context is that disabled people may have experienced a reduction in their income because of their disability. Additionally, it could be argued that disability worsens the psychological disposition towards life, which in turn leads to a less positive assessment of one’s economic circumstances. Transversal studies on this topic do not deal specifically with reverse causality [2,6,14,16-18].

The aim of this paper is to ascertain if the subjective perception of the economic situation of the household is associated with the prevalence of disability in old age net of education level. Our hypothesis is that there will be a statistically significant association with the subjective economic level after adjusting for education.

## Methods

This is a transversal descriptive study based on the Ageing in Spain Longitudinal Study (ELES), Pilot Survey (the database is freely accessible at http://www.proyectoeles.es). Details of the study have been published elsewhere in Spanish [19,20]. Briefly, this was a survey on non-institutionalized people living in Spain aged 50 over. The sample design was polyetapic, with stratification by autonomous community (regions) and size of the city/town of residence of the first stage sampling units (census tracts). Stratification was proportional to the population aged 50 and over living in the strata, although the Basque Country was oversampled. Census tracts were selected randomly with sampling probabilities proportional to their 50 and over population. Second sampling units (households) and informant sampling units (one individual per household) were selected randomly until a proportional representation of strata by gender and decennial age groups was achieved. The sample frame was the commercial household telephone directory, which is commonly used by market research companies in Spain, where census tract is included. The coverage of land telephone lines in Spain between 2004 and 2011 was higher than 92% of households where any resident was aged 50 or over [Spanish National Statistics Institute: personal communication based on the data of the survey on equipment and usage of ICTs in households (Encuesta sobre equipamiento y uso de TIC en los hogares)].

Retrieval of information was arranged sequentially by a computer assisted telephone interview (CATI), nurse visit, computer assisted personal interview (CAPI) and self-administered questionnaire. 1,747 individuals took part in the CATI and 1,400 in the CAPI. Practically all variables for this paper were retrieved by CAPI. The fieldwork was carried out in 2011. The study was approved by the Ethics Subcommittee of the Spanish National Research Council. Informed consent was obtained from all participants.

### Sample for this paper

Our initial intention was to include all individuals who were aged 50 and over, but the inclusion criteria rendered no disability cases before 60 years of age, so being less than 60 years old was an exclusion criterion. Individuals whose disability began before being 50 were excluded because we were interested in disability starting in old age. Interviews answered by a proxy were also excluded because of the subjective nature of the majority of the principal variables.

In order to minimize the main source of reverse causality, where the development of disability can produce a reduction in job income and therefore in the self-perceived economic level, only individuals whose job income levels (including those generated by self-employment) were fixed before becoming disabled were selected: first, retired workers, excluding those whose disability began before they started to receive their retirement pension; second, individuals who were not retired but who were not working at the time the survey was conducted, excluding those whose disability began before they stopped working; and third, individuals who had never worked.

### Statistical analysis

A binary logistic regression was used. The outcome variable was being disabled. We included all those people who answered “I have difficulty” always or sometimes due to a physical or health issue in performing at least one activity of daily living (ADL), either basic (BADLs: getting dressed, including putting on socks and shoes; walking across a room; bathing or showering; eating, including cutting up food; getting out of bed; using the toilet, including standing up and sitting down) or instrumental (IADLs: preparing a hot meal; shopping; making telephone calls; taking medication; doing the housework or gardening; managing money –such as paying bills and keeping track of expenses). If a disability was found to be present, it was asked at which age the disability had appeared. This battery was taken from the English Longitudinal Study on Aging (ELSA) [21].

The principal independent variables were: first, education, which had four categories: less than primary school, primary school completed, secondary school completed, and university degree; second, difficulty in making ends meet, which initially had five categories but which were eventually collapsed to three: with a lot of difficulty and with difficulty, with just enough resources, with ease and with plenty of ease; and third, the self-perceived relative economic situation of the household, which initially was a quantitative variable with range 0–10, however linearity analyses showed a step in the coefficients at the 5–6 score, so the variable was transformed to a dichotomous one (0 to 5 points and 6 to 10 points). The adjustment of the model after this transformation was improved.

The covariates were: gender and age (in years). In order to assess psychological disposition as a confounder and a possible source of reverse causality, we used the question “How often do you think that benefits make amends for the adversities of living?” There were five initial response categories: hardly ever, few times, sometimes, quite a lot of times, almost always [22]. To reduce the number of variables in the multivariable models, the first three categories and the last two were collapsed, producing the final categorization of bad and good disposition. As mediators between socioeconomic variables and disability, two risk behaviours were used. The first was weekly alcohol consumption, which had three categories: below 280 g in men and 170 g in women, above these quantities, and former drinkers (people who used to drink alcohol but had given up in the last 12 months). The second was smoking, which had three categories: never smoker, former smoker, current smoker.

Analysis strategy used: Bivariate analyses were carried out between outcome and predictors, among subjective economic variables and between subjective economic variables and education. Two sets of logistic models were built: one for the variable “difficulty in making ends meet” and another for “self-perceived relative economic position of the household”. In each set, first, each independent variable was tested alone. Secondly, gender and age were added to the model. Thirdly, all independent variables, except psychological disposition, were introduced at the same time. At this stage, an interaction term between age and subjective economic situation variables was added. Fourthly, a reduced model was obtained, where non-significant (p ≥ 0.05) variables (those whose elimination did not change the coefficients of the other variables in more than 10% and did not increase their standard errors) were taken away [23]. Fifthly, the variable of disposition was incorporated into the multivariable models to ascertain confounding and reverse causality issues. Lastly, alcohol and tobacco consumption variables were added. Requirements for logistic models were checked and complex sample design features were accounted for. Analyses were carried out with the statistical package Stata 12.1 [24].

## Results

### Participation

**Figure 1:**
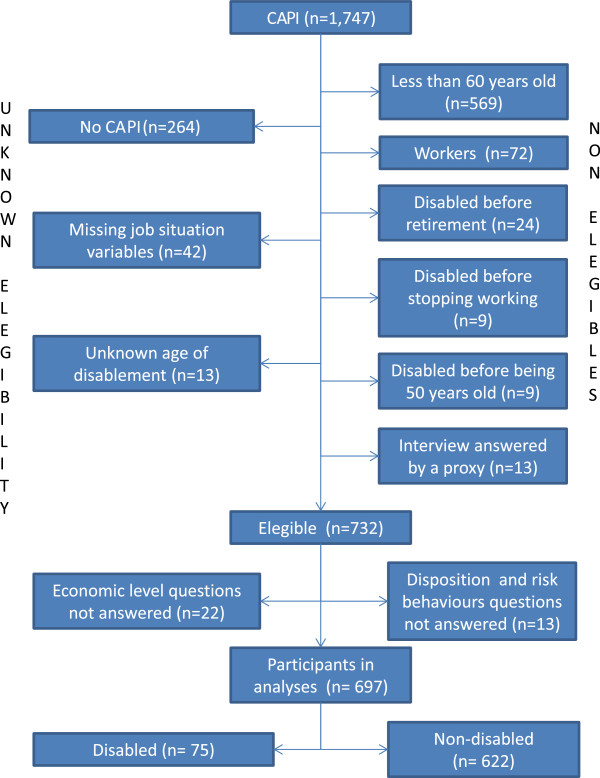
**Study participation flow.** CAPI: Computer Assisted Personal Interview.

This is shown in Figure 1. Only 4.7% of those eligible have missing values, so they can be dropped from the analyses without biasing the results. Nevertheless, the eligibility status could not be ascertained in 18.3% of the survey participants, including 264 subjects who did not take part in the CAPI questionnaire, where most of the variables for this paper were included.

Characteristics of the final 697 participants are shown in Table 1. The mean age is 72 years of age and 130 individuals are aged 80 or over; 56% are women. Almost half have attended less than primary school. 17.6% have difficulties making ends meet. More than half score their household economic situation above six.

**Table 1:** Characteristics of the sample and bivariate analyses of disability and predictors and subjective economic variables

	**Total (n = 697)**	**Non-disabled (n = 622)**	**Disabled (n = 75)**	**p**	**Make ends meet…**			**p**	**Dwelling score**		**p**
					**With difficulty (n = 109)**	**Only slightly (n = 282)**	**Easily (n = 306)**		**0-5 (n = 288)**	**6-10 (n = 409)**	
Mean age 95% confidence interval	71.9 (71.1-72.6)	71.3 (70.6-72.0)	76.3 (74.3-78.4)	<0.001							
% of women	55.9	53.2	77.1	<0.001							
% with less than primary school	46.5	44.1	65.1	0.002	64.5	52.2	34.0	<0.001	56.8	38.9	<0.001
% who have finished primary school	22.2	22.5	19.7		21.7	25.5	19.5		23.9	21.0	
% who have finished secondary school	12.2	12.6	8.5		7.5	8.0	17.8		8.9	14.5	
% with a university degree	19.1	20.7	6.7		6.3	14.3	28.7		10.4	25.6	
% who makes ends meet…easily	43.3	45.8	22.9	<0.001							
Only slightly	39.1	38.7	42.6								
With difficulty	17.6	15.5	34.4								
% who score their household 6 to 10	57.7	61.0	31.5	<0.001	18.8	52.1	78.5	<0.001			
% with bad psychological disposition	31.7	29.2	51.7	<0.001							
% of never smokers	60.1	59.2	74.4	0.039							
Former smokers	28.9	29.7	22.4								
Current smokers	10.3	11.2	3.2								
% of drinkers below risk levels	84.2	85.9	70.6	<0.001							
Drinkers above risk levels	6.9	7.1	5.3								
Former drinkers	8.9	7.0	24.0								

ns for unweighted sample, percentages for weighted sample.

### Bivariate analyses

As shown in Table 1, those with difficulties in carrying out daily life activities are older and more frequently women and have a lower education level and more difficulties in making ends meet. They score the economic situation of their household above six less frequently.

Education and subjective economic situation are associated in the expected way (Table 1): the higher the education level, the better the economic situation. Both subjective economic variables are also related in the expected direction (Table 1): 78.5% of those who make ends meet easily score their household economic situation above six, compared to 18.8% of those who experience difficulty.

Smoking is associated with disability, but not in the expected direction: those who are non-disabled are more frequently smokers and less frequently never smokers compared to those who are disabled. Former drinkers are much more prevalent among disabled individuals and drinkers below risk levels are more common among those who are non-disabled.

Bivariate logistic regressions confirm the results (first columns of Tables 2 and 3): there is a gradient in the association of disability with different levels of difficulty in making ends meet. Making ends meet with difficulty multiplies by 4.44 (confidence interval at the 95% level –CI 95%- 2.24-8.83) the odds of being disabled, a greater odds ratio than that of being a woman (odds ratio = 2.96 -CI 95% 1.69-5.17). The odds ratio for scoring the economic level of the household below six is of the same magnitude (3.40 –CI 95% 2.11-5.49). There is a clear protective gradient in the odds ratios of the different education levels: 0.59 (CI 95% 0.32-1.11) for primary school, 0.46 (CI 95% 0.19-1.09) for secondary school and 0.22 (0.09-0.56) for university degree, where the category “less than primary school” is given the reference value of one.

**Table 2:** Bivariate and multivariate analyses of disability and making ends meet

**Some difficulty in ADLs**	**Bivariate models**			**Models adjusted for age and gender**			**Multivariable model**			**Multivariable model adjusted for disposition**			**Multivariable model adjusted for disposition, smoking and drinking**		
	**OR**	**CI 95%**	**p**	**OR**	**CI 95%**	**p**	**OR**	**CI 95%**	**p**	**OR**	**CI 95%**	**<0.001**	**OR**	**CI 95%**	**<0.001**
For each additional year of age	1.10	(1.06-1.14)	<0.001				1.10	(1.05-1.14)	<0.001	1.10	(1.05-1.15)	<0.001	1.10	(1.05-1.15)	<0.001
Women (ref. men)	2.96	(1.69-5.17)	<0.001				2.47	(1.33-4.58)	0.004	2.34	(1.26-4.36)	0.007	2.86	(1.32-6.19)	0.008
Primary school (ref. less than primary)	0.59	(0.32-1.11)	0.011	0.80	(0.40-1.59)	0.217	0.88	(0.43-1.79)	0.521	0.84	(0.41-1.72)	0.520	0.92	(0.46-1.84)	0.767
Secondary school (ref. less than primary)	0.46	(0.19-1.09)		0.69	(0.29-1.65)		0.90	(0.37-2.17)		0.87	(0.35-2.14)		0.99	(0.37-2.64)	
University (ref. less than primary)	0.22	(0.09-0.56)		0.35	(0.13-0.93)		0.48	(0.18-1.29)		0.49	(0.19-1.27)		0.61	(0.23-1.63)	
Make ends meet only slightly (ref. easily)	2.20	(1.10-4.42)	<0.001	1.79	(0.86-3.72)	0.001	1.68	(0.81-3.46)	0.003	1.49	(0.72-3.10)	0.013	1.57	(0.74-3.31)	0.010
Make ends meet with difficulty (ref. easily)	4.44	(2.24-8.83)		3.90	(1.87-8.12)		3.45	(1.68-7.08)		3.02	(1.41-6.48)		3.20	(1.46-7.03)	
Bad psychological disposition (ref. good)	2.60	(1.54-4.49)	<0.001							2.22	(1.21-4.09)	0.011	2.42	(1.27-4.62)	0.008
Former smokers (ref. never smokers)	0.60	(0.32-1.13)	0.051										1.40	(0.59-3.31)	0.246
Current smokers (ref. never smokers)	0.23	(0.05-0.97)											0.43	(0.10-1.79)	
Drinkers above risk levels (ref. below)	0.91	(0.28-2.91)	<0.001										1.90	(0.59-3.31)	<0.001
Former drinkers (ref. below risk levels)	4.18	(2.18-7.99)											4.63	(2.16-9.92)	
Model p value						<0.001			<0.001			<0.001			<0.001
Hosmer & Lemeshow p value									0.598			0.112			0.348

n = 697. CI: Confidence interval. ADL: Activities of daily living. Ref: Reference category.

**Table 3:** Bivariate and multivariate analyses of disability and self-perceived economic situation of the household

**Some difficulty in ADLs**	**Bivariate models**			**Model adjusted for age and gender**			**Full multivariable model**			**Reduced multivariable model adjusted for disposition**			**Reduced multivariable model adjusted for disposition, smoking and drinking**		
	**OR**	**CI 95%**	**p**	**OR**	**CI 95%**	**p**	**OR**	**CI 95%**	**p**	**OR**	**CI 95%**	**p**	**OR**	**CI 95%**	**p**
For each additional year of age	1.10	(1.06-1.14)	<0.001				1.10	(1.06-1.15)	<0.001	1.10	(1.06-1.15)	<0.001	1.10	(1.06-1.15)	<0.001
Women (ref. men)	2.96	(1.69-5.17)	<0.001				2.87	(1.56-5.31)	0.001	2.85	(1.58-5.16)	0.001	3.32	(1.51-7.28)	0.001
Primary school (ref. less than primary)	0.59	(0.32-1.11)	0.011	0.80	(0.40-1.59)	0.217	0.83	(0.40-1.69)	0.462						
Secondary school (ref. less than primary)	0.46	(0.19-1.09)		0.69	(0.29-1.65)		0.89	(0.37-2.17)							
University (ref. less than primary)	0.22	(0.09-0.56)		0.35	(0.13-0.93)		0.47	(0.18-1.23)							
Household economic situation score below 6 (ref. equals or above 6)	3.40	(2.11-5.49)	<0.001	3.60	(2.18-5.93)	<0.001	3.37	(2.04-5.56)	<0.001	3.25	(1.93-5.47)	<0.001	3.30	(1.92-5.68)	<0.001
Bad disposition (ref. good)	2.60	(1.54-4.49)	<0.001							2.13	(1.15-3.95)	0.017	2.38	(1.24-4.56)	0.009
Former smokers (ref. never smokers)	0.60	(0.32-1.13)	0.051										1.33	(0.56-3.12)	0.233
Current smokers (ref. never smokers)	0.23	(0.05-0.97)											0.40	(0.09-1.82)	
Drinkers above risk levels (ref. below)	0.91	(0.28-2.91)	<0.001										2.23	(0.65-7.66)	<0.001
Former drinkers (ref. below risk levels)	4.18	(2.18-7.99)											4.80	(2.17-10.63)	
Model p value						<0.001			<0.001			<0.001			<0.001
Hosmer & Lemeshow p value									0.510			0.405			0.308

n = 697. CI: Confidence interval. ADL: Activities of daily living. Ref: Reference category.

### Multivariate analyses

#### a) Models for difficulty in making ends meet

Results are shown in Table 2. In the model adjusted for age and gender, the odds ratios of level of studies are closer to one compared to the bivariate model and they lose significance (0.80 –CI 95% 0.40-1.59- for primary school, 0.69 –CI 95% 0.29-1.65- for secondary school and 0.35 –CI 95% 0.13-0.93- for university degree; p = 0.217). Those of economic difficulties are reduced but keep their significance. In the multivariable model, level of studies remains, although it is not significant, because of its confounding effect. Having difficulties making ends meet multiplies the odds of being disabled by 3.45 (CI 95% 1.68-7.08) compared to those without difficulties, still a greater value than that of being a woman compared to a man (2.47 CI 95% 1.33-4.58). Making ends meet only slightly multiplies the odds of being disabled by 1.68 (CI 95% 0.81-3.46). The interaction between age and difficulty in making ends meet is non-significant (p = 0.589). Further adjustment for psychological disposition reduces the odds ratios even more but the variable remains statistically significant (p = 0.013). Adding smoking and drinking variables does not modify the associations notably (having difficulties 3.20 –CI 95% 1.46-7.03-; making ends meet only slightly 1.57 –CI 95% 0.74-3.31). In the adjusted models, the direction of the bivariate association of former smoking with disability is reversed, while that of current smoking is reduced. The marginal statistical significance of the bivariate analysis is lost. The relationship of alcohol consumption and disability remains significant after adjustment: the odds ratio for former drinkers is increased and that for current drinkers changes direction.

**Figure 2:**
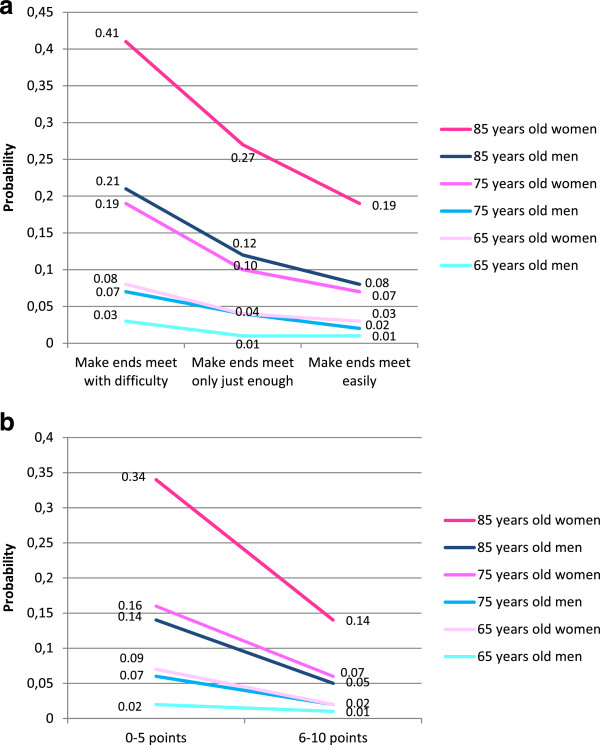
**Probability of disability predicted by the multivariate models adjusted for psychological disposition and health risk behaviours.** 2**a**. For different levels of difficulty in making ends meet. 2**b**. For different self-perceived household economic scores.

The coefficients of the multivariate model adjusted for disposition and risk behaviours allow us to calculate the probability of being disabled for individuals with different attributes. Probabilities are presented for individuals with less than primary studies, with a good psychological disposition, who do not smoke and drink below risk levels, with varying combinations of age, gender and economic perceptions. They are between two and three times higher among those experiencing difficulties in making ends meet compared to those without difficulties, depending on gender and age (Figure 2a). 75 and 85 year old men have almost the same probabilities as women who are ten years younger. From a different perspective, the probability of being disabled for a man with difficulties in making ends meet at all ages is similar to that of a woman of the same age or a man ten years older without economic difficulties. At ages 75 and 85, a woman without economic difficulties has a similar probability of being disabled to that of a woman ten years younger with economic difficulties.

#### b) Models for self-perceived economic situation of the household

Results are shown in Table 3. In the model adjusted for age and gender the association of the subjective economic situation of the household with disability is slightly increased (3.40 –CI 95% 2.11-5.49 vs 3.60 –CI 95% 2.18-5.93). This is the final reduced model, because education has no confounding effect and thus may be removed. Again the odds ratio of the economic situation of the household is greater than that of being a woman (compared to being a man: 2.96, CI 95% 1.69-5.17). The interaction between age and self-perceived economic situation is non-significant (p = 0.299). Adjusting this model for psychological disposition reduces the association of the economic situation, but the variable remains statistically significant (p < 0.001). Further adjustment for health risk behaviours does not modify the odds ratio (3.30 –CI 95% 1.92-5.68).

In probability terms (Figure 2b), a 65 year-old woman scoring her household below six has a four times higher probability of being disabled compared to a woman of the same age scoring above six. The probabilities are tripled for 75 or 85 year-old men and doubled for 75 or 85 year-old women and 65 year-old men. At all ages, a man who scores his household low has a similar probability of being disabled to a woman of his same age or a man ten years older who scores her/his household high. At 75 and 85 years of age, a woman who scores her household high has a probability of being disabled similar to that of a woman ten years younger who scores her household low.

## Discussion

In the Spanish population over 60 years of age, the subjective household economic situation measured in two different ways is strongly and consistently related with the prevalence of disability net of age, gender and education level. After adjusting for gender and age, education level is no longer associated with disability. The predicted probabilities calculated by the models are dramatic: having economic difficulties has the same effect on disability prevalence as being 10 years older or being a woman instead of a man.

We confirm our hypothesis that the self-perceived economic situation of the household is associated with prevalent disability over and above the education level of the individual. A similar result has been found for prevalence of limitations in basic activities of daily living (BADL) among the elderly in Hong Kong [17] and instrumental activities of daily living (IADL) among the adult population in the United States [2]. An association of a similar variable with incident disability for BADL and IADL has been reported among the elderly in Shanghai [11]. Nevertheless, this association was not found with BADL disability incidence among the elderly in China [10]. An association of financial satisfaction with bad self-rated health, depressive symptoms and mortality was also found among the Taiwanese elderly [15]. Moreover, it has been shown that financial strain predicts better than income the prevalence and incidence of elderly disability for BADLs [18] and elderly women’s mortality [25].

The improvement in prediction provided by the subjective economic perception of income compared to that based on income alone could be due to the following. First, it captures unobserved dimensions of current economic position that are missed when measuring income, for example, disposable income. Second, it encompasses psychological factors related to how the individual is managing his/her economic situation. Third, difficulties in managing financially could be due to low morale and cognitive impairment [18]. Fourth, individuals with a more positive psychological disposition will score their economic position and health higher [26], whereby psychological disposition would be a confounder. Fifth, psychological attitude may act as an intermediary construct in the path that leads from bad economic situation to disability [27]. Finally, it could even be a source of reverse causality, whereby disability worsens psychological attitude, which in turn leads to a worse evaluation of the personal economic situation.

Either of the first two mechanisms would justify our finding that some individuals with high incomes perceive their economic circumstances as not good. This is related to a lack of a common reference for all interviewees, but also, as explained by Mau [28], to the fact that the perception of the country’s overall economic circumstances may also influence individual economic assessment. The third explanation could be invoked by our variable of making ends meet, but not the relative position of the household variable. To discard the fourth, fifth and sixth explanations, we have adjusted our multivariable models by a variable measuring will to live, a positive aspect of psychological attitude [29]. After this adjustment, the association of the subjective economic variables with disability is reduced, but it does not disappear. Therefore it cannot be entirely explained by confounding or intermediary effects. These results do not support reverse causality based on psychological pathways as an alternative explanation, either. In fact, the change in coefficients is modest. This can be understood in terms of the reported findings that optimism, another positive aspect of psychological attitude, is minimally related to socioeconomic status [27], and that disposition is less associated with subjective health than objective measures of health [30].

Apart from their intrinsic advantages, subjective economic variables are not exposed to selection bias because of missing values, unlike income. In our study, of the total unweighted sample, 92 individuals (13%) did not declare their household income. This proportion is larger among those living in households in better economic circumstances, especially when this is ascertained through difficulty in making ends meet (data not shown). Others have found also that those better off tend to underreport their income [13]. In much previous research, details on missing data on income are absent [2-4], which makes it difficult to judge the existence of bias.

We have not found evidence of a differential association of subjective economic variables with disability at different ages, as others have [18]. These associations are not explained by two health risk behaviours. Notably, the introduction in the model of smoking and drinking variables reduces the association with education level, which therefore appears more related to these risk variables than income. Adjustment for sociodemographic variables changes the unexpected association of former smoking and excess drinking with less disability, which is a proof of age and gender confounding: drinking and smoking, including former smoking, is more frequent among younger old men, who have less disability. However, adjustment reduces the unexpected association of current smoking with less disability only partially. Reverse causality may be playing a role: those with no disease and therefore no disability keep on smoking. Reverse causation has been described for ex-smokers [31], but we have not found any reference to reverse causality in current smokers in relation to elderly disability. In any case, results for smoking are not statistically significant.

In our hypothesis, it was not anticipated that education level would lose its association after adjusting for age and gender. In previous research, the association of education with health variables is usually reduced after adjusting for income, but not for sociodemographic variables [1,2,6,7,9,14,16,32],[33]. We have found one study where education was not associated with disability for BADL prevalence among the elderly in Hong Kong [17] and another one where it lost its association with disability for BADL incidence (but not for IADL) among the elderly in China after adjusting for age and gender [8]. Our interpretation is that education level is so associated with age and gender among the elderly populations in Spain and China that it is impossible to disentangle their particular effects. For example, in the 60 to 69 year olds of our sample, 34.3% have attended less than primary school and 21% university, compared to 60.8% and 14.6% respectively of those aged 80 and over. Among women, 53.9% have attended less than primary school and 13% university, compared to 37.1% and 25.9% of men, respectively. This is the result of historical developments in access to schooling of the population.

Our strengths are as follows. First, we have worked with a sample designed to be representative of the elderly population in Spain. Second, there was a very high response in all variables among those eligible. Third, reverse causality was reduced through the selection of individuals whose incomes were fixed before they developed disabilities. Nevertheless, limitations in sample size have precluded us from excluding individuals who developed their disability very early after retirement, and who were probably receiving lower pensions because of lower earnings due to bad health during their working life. In addition, we have not been able to exclude those who have experienced an increase in expenses, and therefore a reduction in disposable income, after disability appeared.

There are some other limitations in our study. We could not establish eligibility for a large percentage of the original sample, mainly because they did not take part in the phase of the survey where most of the variables we were interested in were collected. Those not taking part were older and less educated [19]. This may have contributed to lessening the association of the economic situation and education with disability, ultimately making the latter non-significant. However, we would not expect selection bias to interfere with our principal hypothesis. Because of sample size limitations, we could not check the existence of the interaction between education and economic level that others have found [15,32], nor between socioeconomic variables and gender. Also because of the reduced sample size, we have not been able to deal with the fact that some men may have reported limitations in some instrumental activities (preparing a hot meal, shopping, housework) more due to a gender segregated distribution of activities than to health problems. For the same reason, we have had to expand our definition of disability to those who have difficulties “sometimes”, who are less disabled. This would introduce some misclassification bias that would reduce, not increase, the chances of finding significant results. Mainly because of low response rates to other parts of the questionnaire, we have been able to adjust our models for some health risk behaviours only. Other variables (body mass index, diet) could have shown a different effect.

## Conclusions

Our results suggest that the self-perceived economic situation of the household is a better predictor of prevalent disability than the education level of the individual. As the economic circumstances of the elderly are much easier to improve than their formal education, this finding supports feasible interventions which could lead to a reduction in the prevalence of disability. These measures could take the form of increasing monetary support, at least for the humblest households, or reducing their expenses by means of free drug prescriptions or discounts in the prices of transport and utilities [25]. Before proposing them as effective public health policies, quantitative and qualitative studies exploring the mismatch between subjective and objective economic variables and intervention studies showing the effect of the proposed interventions on the development of and recovery from disability are warranted.

## Competing interests

The authors declare they have no competing interests.

## Authors’ contributions

AR and AB conceived and designed the study. AR performed the statistical analyses and drafted the manuscript. AB, MS, RP, IM and PD made relevant contributions to the analysis and to the manuscript. All authors read and approved the final manuscript.

## Pre-publication history

The pre-publication history for this paper can be accessed here:

http://www.biomedcentral.com/1471-2318/14/60/prepub
